# Predictors of reproductive and non-reproductive outcomes of gonadotropin mediated pubertal induction in male patients with congenital hypogonadotropic hypogonadism (CHH)

**DOI:** 10.1007/s40618-021-01556-x

**Published:** 2021-03-18

**Authors:** B. Cangiano, G. Goggi, S. Federici, C. Bresesti, L. Cotellessa, F. Guizzardi, V. Vezzoli, P. Duminuco, L. Persani, M. Bonomi

**Affiliations:** 1 Department of Medical Biotechnology and Translational Medicine, University of Milan, Milan, Italy; 2 Department of Endocrine and Metabolic Diseases and Laboratory of Endocrine and Metabolic Research, IRCCS Istituto Auxologico Italiano, P.le Brescia 20, 20149 Milan, Italy

**Keywords:** Isolated hypogonadotropic hypogonadism, GnRH deficiency, Puberty, Androgenization, Genetics

## Abstract

**Purpose:**

To investigate predictors of testicular response and non-reproductive outcomes (height, body proportions) after gonadotropin-induced puberty in congenital hypogonadotropic hypogonadism (CHH).

**Design:**

A retrospective analysis of the puberty induction in CHH male patients, undergoing an off-label administration of combined gonadotropin (FSH and hCG).

**Methods:**

Clinical and hormonal evaluations before and during gonadotropin stimulation in 19 CHH patients genotyped by Targeted Next Generation Sequencing for CHH genes; 16 patients underwent also semen analysis after gonadotropins.

**Results:**

A lesser increase in testicular volume after 24 months of induction was significantly associated with: (I) cryptorchidism; (II) a positive genetic background; (III) a complete form of CHH. We found no significant correlation with the cumulative dose of hCG administered in 24 months. We found no association with the results of semen analyses, probably due to the low numerosity. Measures of body disproportion (eunuchoid habitus and difference between adult and target height: deltaSDSth), were significantly related to the: (I) age at the beginning of puberty induction; (II) duration of growth during the induction; (III) initial bone age. The duration of growth during induction was associated with previous testosterone priming and to partial forms of CHH.

**Conclusions:**

This study shows that a strong genetic background and cryptorchidism, as indicators of a complete GnRH deficiency since intrauterine life, are negative predictors of testicular response to gonadotropin stimulation in CHH. Body disproportion is associated with a delay in treatment and duration of growth during the induction, which is apparently inversely related to previous androgenization.

**Supplementary Information:**

The online version contains supplementary material available at 10.1007/s40618-021-01556-x.

## Introduction

CHH is a rare disease with a strong genetic background, which leads to absent (complete forms) or incomplete (partial forms) puberty due to insufficient hypothalamic-pituitary stimulation of the gonads [1–3]. The treatment of this condition is aimed to restore the pubertal process, achieve normal sex hormones, sexual life and, possibly, fertility. Testosterone administration is the most common treatment for puberty induction in male CHH patients, but this approach fails to stimulate testicular growth and functions, which may have physical, psychological and reproductive consequences. GnRH or gonadotropin-mediated induction of puberty can support testicular growth and functions thus representing a “true” replacement therapy probably leading to additional beneficial effects other than virilization [3].

However, data concerning the gonadotropin-mediated pubertal induction are scarce, as the available studies are all characterized by low numerosity, heterogeneous age of the enrolled patients, mixed underlying causes (series composed of CHH and combined pituitary hormone deficiencies, CPHDs), variable durations and schemes of treatment as well as pre-existing conditions [4–12]. The extremely different results obtained in all these studies are therefore not surprising. Only two of four randomized clinical trials were not performed in adults and did not evaluate genetics or non-reproductive outcomes of the treatment [13–16].

To better understand the effects and relevance of the “off-label” gonadotropin induction of puberty, we retrospectively analyzed a series of CHH patients with the aim to evaluate (I) clinical and biochemical predictors of the testicular response, (II) the non-reproductive outcomes of the therapy (height, body proportions, penis length) and their determinants, and (III) the influence of changes in therapeutic schemes and dosages.

## Patients and methods

### Patients

We revised data from 19 male patients with CHH in which an off-label gonadotropin-mediated induction of puberty was started between the ages of 14 and 23 years. The off-label use of gonadotropins for puberty induction in CHH had been included in the official protocol for diagnosis and care of patients with such rare disease approved by the Regional Health System in 2010 (http://malattierare.marionegri.it/content/view/69/). After approval of the Institutional Review Board (Istituto Auxologico Italiano, 05C202), all parents or adult patients gave the informed consents for: (a) off-label gonadotropin therapy; (b) use of the anonymized clinical, biochemical and genetic data for research and publication purposes. At diagnosis, all subjects had no puberty onset after the age of 14 or showed the failure to complete puberty after the age of 16, associated with low total testosterone (*T* ≤ 3.5 nMol/L) and inappropriately low/normal gonadotropins. Those without a genetic diagnosis, had no puberty onset by 18 years old, or showed an interruption of the pubertal process, or had anosmia and/or other indicative hallmarks such as familiarity for CHH/KS. All the patients were confirmed to have persistent hypogonadotropic hypogonadism after completing the induction of puberty.

Serum LH, FSH, estradiol, testosterone concentrations were measured by electrochemiluminescence immunoassay ‘ECLIA’ from Roche Diagnostic (Roche Diagnostics GmbH). All patients with secondary causes of hypogonadism were excluded: each patient was negative for other pituitary hormonal defects, hyperprolactinemia, structural alterations at hypothalamic-pituitary MRI with contrast, and for hemochromatosis. Likewise, no subject had other previous or present pathology, neither previous chemo- or radiotherapy treatments.

All were subjected to radiological study of the olfactory structures; an olfactory functional test was performed using the Brief Smell Identification Test (BSIT Sensonic, NJ, USA). Abdominal US for the study of renal agenesis/hypoplasia and audiometry when a hearing defect was suspected, were also performed. In 12 patients, bone age was studied performing a wrist and left-hand radiography, and biological (bone) age was calculated using the TW3 (Tanner–Whitehouse 3) method. In compliant adult patients at the end of pubertal induction, a semen analysis (SA) was obtained. Bi-testicular volume (BTV) was calculated as the sum of the volumes of the two testicles, measured using Prader orchidometer.

Patients were divided according to BTV, between those with totally absent (BTV < 8 mL) pubertal development and those with partial forms of CHH (BTV ≥ 8 by 14 years and without progression in the last 12 months). Data collected also included physical examination and history of unilateral or bilateral cryptorchidism. To identify possible eunuchoid proportions an arm span > 5 cm greater than the height of the subject was considered indicative of body disproportion (eunuchoid habitus).

Each patient’s growth was evaluated using ethnicity-adjusted percentiles: in Caucasian and Italian subjects Cacciari’s curves and centiles were used, whereas in the four non-Italian boys Tanner curves were selected. For each subject height, weight, BMI, growth rate, target height (TH) were reported both as normal values and in standard deviations scores (SDS); the difference between adult height SDS and TH SDS was also reported (deltaSDSth). The variations in height at each visit were calculated in SDS (referred in this manuscript as SDS height change).

Curves for all the parameters were generated using SIEDP (Società Italiana di Endocrinologia e Diabetologia Pediatrica) Growth Calculator (version 3).

Duration of growth during puberty induction was calculated as the number of days lapsed from the beginning of gonadotropin therapy to the visit in which growth rate dropped below 2 cm/year. The methods and reference values used for SA were taken from the latest edition of the WHO manual (2010) [17]. Table 1 shows the characteristics of the patients.

**Table 1:** Characteristics of patients studied

Patient	Prev. TE therapy	Diagnosis BTV (mL)	Peak LH to GnRH test (U/L)	BMI SDS	Bone age (y)	Chronological age (y)	Cryptorchidism	FSH pre-treatment	Genes involved	Variants found	Genetic cause/inheritance	Olfactory MRI	Smell test
1	No	2	0.8	− 0.14	n.d	17.8	2	Yes	PROKR2 FEZF1	p.L173R p.K128Q	Explained/ oligogenic	aplB/T hypoplS	Anosmia
2	Yes	10	26.5	0.13	14	15.4	0	No	FGFR1	p.R822C	Explained/ AD	Normal	Normal
3	No	3	0.5	1.89	13.3	16.1	1	Yes	ANOS1	p.V182AfsX3	Explained/ hemizygosis	aplB/T/S	Anosmia
4	No	7	2.5	1.63	15.1	16.7	0	Yes	GnRH	p.Q106R	Unexplained	aplBT/hypoplS	Normal
5	Yes	12	n.d	1.03	16.4	20.4	0	No	0	–	Unexplained	aplB/T hypoplS	Anosmia
6	Yes	2	4.6	1.02	13.5	13.0	0	Yes	FGFR1	p.G402WfsX6	Explained/ AD	apl B	Anosmia
7	Yes	4	0.3	0.72	15	18.6	0	Yes	GnRHR homo	p.N12IfsX11 p.N12IfsX11	Explained/ homozygosis	Normal	Normal
8	Yes	7	0.07	0.78	15.6	20.8	0	No	0	–	Unexplained	Normal	Normal
9	No	4	0.5	0.74	12.9	16.1	0	Yes	0	–	Unexplained	Normal	Normal
10	No	12	n.d	2.88	n.d	23.8	0	Yes	0	–	Unexplained	hypoplB/T	Anosmia
11	No	5	0.4	− 0.09	n.d	19.9	0	Yes	CHD7	p.R758C	Explained/ AD	Normal	Normal
12	No	4	0.6	− 1	13	14.9	2	Yes	0	–	Unexplained	aplB/T/S	Anosmia
13	Yes	8	6.7	0.01	n.d	19.9	2	No	0	–	Unexplained	Normal	Normal
14	No	5	4.2	1.36	15.2	17.6	2	Yes	PROKR2	p.R268H	Unexplained	hypopl B/S	Normal
15	No	5	0.5	− 0.27	14.6	17.9	1	Yes	SEMA3A	p.R637H	Unexplained	Normal	Normal
16	No	6	1.1	0.26	12	14.6	0	Yes	0	–	Unexplained	Normal	Normal
17	Yes	3.5	0.8	− 0.25	n.d	17.2	2	No	0	–	Unexplained	Normal	Normal
18	No	6	12.4	1.29	14	14.4	0	No	0	–	Unexplained	Normal	Normal
19	No	2	3.5	1.17	n.d	14.7	2	Yes	SEMA3AFGF8	p.N153S p.G33V	Explained/ oligogenic	aplB/ hypoplT	Anosmia

*Te* testosterone, *AD* autosomal dominant, *apl* aplasia, *hypopl* hypoplasia, *B* olfactory bulbs, *T* olfactory traits, *S* olfactory sulci, *BTV* bi-testicular volume. Cryptorchidism 1: unilateral, 2: bilateral

### Genetic analysis by targeted next-generation sequencing (NGS)

Each patient underwent a genetic investigation, using a targeted NGS technique, to look for rare allelic variants. We extracted the genomic DNA of each patient from peripheral blood lymphocytes using Gene Catcher gDNA 96 _ 10 mL Automated Blood kit (Invitrogen, Life TechnologiesTM, Carlsbad, CA, USA). The CHH gene panel was designed using Illumina Design Studio (San Diego, CA, USA) and included the following CHH candidate genes: ANOS1, FGFR1, PROKR2, PROK2, GNRHR, GNRH1, GNRH2, KISS1, KISS1R, TAC3, TACR3, HS6ST1, FGF8, CHD7, DUSP6, FEZF1, FGF17, FLTR3, IL17, SEMA3A, SEMA3E, SEMA7A, SOX2, SOX10, SPRY4, WDR11, HESX1, NELF. The 28 CHH genes assessed for the purposes of this study were consistently represented in all sequence capture panels. Libraries were prepared using Illumina Nextera Rapid Capture Custom Enrichment kits according to the manufacturer’s protocols. All regions not correctly sequenced were recovered with NexteraVR DNA Library Preparation kit (Illumina, San Diego, CA, USA). For subsequent analyses, we included as “rare variants” [18, 19] all known pathogenic, or rare non-synonymous or splicing-site variants (Minor Allele Frequency, MAF ≤ 0.01) and novel non-synonymous or splicing-site variants. The prevalence and the functional annotation of the identified variants were checked in public and licensed databases (Ensembl, UCSC Genome browser, 1000 Genome project, ExAC Browser, NCBI, HGMD professional), considering the ethnic groups (Europeans). Each variant found was confirmed by Sanger direct sequencing using BigDyeVR Terminator v.3.1 Cycle Sequencing Kit (Life Technologies, Carlsbad, CA, USA) on a 3100 DNA Analyzer from Applied Biosystems (Foster City, CA, USA). To study patients with a positive genetic background (not necessarily already validated) we performed the evaluation both comparing patients harboring at least one CHH-related variant with wild-type patients and comparing those having a hemizygous or homozygous genotype with the others.

### Puberty induction

In all the patients enrolled gonadotropin-mediated induction of puberty was obtained using urofollitropin (FSH, Fostimon) and hCG (Gonasi HP) with a weekly dosage divided in a scheme of 2 or 3 subcutaneous injection. Thirteen patients underwent a preliminary FSH administration (75 IU three times per week) of 4 months before the combination with hCG treatment. Six patients had already undergone a testosterone priming (intended as low-dose short-term treatments for differential diagnosis [20]) before coming to our attention. The majority of subjects began the treatment with FSH 75 IU 3 times/week and hCG 250 IU 2 or 3 times/week. Total hCG administered during the induction (cumulative hCG dose) was intended as the sum of the cumulative doses of each period. The cumulative dose of a period was calculated multiplying the weekly dosage of Gonasi HP for the number of weeks of each interval intercurrent between the visits. In Supplementary Table 1 it is reported the standard scheme of treatment.

### Statistical methods

Statistical analysis was performed using SPSS, version 21.0, statistical package (SPSS Inc., Chicago, IL, USA). To evaluate the determinants of the response to gonadotropins, the data obtained were analyzed using Mann–Whitney test and multiple linear regressions to compare both BTV after 18 and 24 months of gonadotropin-mediated pubertal induction and parameters obtained from semen analysis (SA) with pre-treatment with FSH, pre-treatment with testosterone, presence of cryptorchidism at birth, partial *vs.* complete CHH forms, the cumulative hCG dose administered in two years, presence of CHH gene variants *vs.* wild-type (WT), homozygous and hemizygous genotypes vs other genotypes (heterozygous and WT). Similarly, to evaluate the non-reproductive consequences of the induction with gonadotropins, eunuchoid habitus, final penile length and deltaSDSth were studied in their association with age at the beginning of the induction therapy, bone age at the beginning of the treatment, cumulative 2 years hCG administration, and duration of growth during induction (calculated as time lapsed from the beginning of the treatment and the moment when growth rate drops below 2 cm/year).

We also evaluated the determinants of the duration of growth during induction, studying with multiple linear regressions its association with pre-therapy BTV, age at the beginning of the induction, previous testosterone treatment, pre-treatment with FSH, partial CHH forms. To find out the parameters associated with gonadotropin dosage we used different Kruskal–Wallis tests.

## Results

The characteristics of our cohort are summarized in Table 2. Results of SA and BTV at 18 and 24 months are reported in Table 3.

**Table 2:** Characteristics of the CHH patients included

	Mean (SD)
BMI, SDS	+ 0.7 (± 0.9)
RUS bone age, years	14.2 (± 1.25)
Age of induction of puberty, years	17.4 (± 2.7)
BTV at diagnosis, mL	5.3 (± 2.7)
Complete forms according to BTV at diagnosis	84%
LH peak to GnRH test, U/L	3.9 (± 6.6)
Cryptorchidism [bilateral]	42 [31] %
Pretreated with FSH	68%
Previous priming with T	37%
Duration of previous T treatment, months	9 (± 9.7)

*RUS*  radial-ulnar-short bones, *BTV* bi-testicular volume, *T*  testosterone

**Table 3:** Reproductive outcomes of induction therapy

	18th month BTV (mL)	24th month BTV (mL)	SA Volume (mL)	SA Concentration (nx10^6^/mL)	SA total count 10^6^	SA motility (%)	SA normal forms (%)
1	9	10					
2	38	42	2.7	42	113	44	2
3	9	12	1	0.0001	0.0001	0.0001	0.0001
4	26	36	1	19	19	39	1
5	24	26	3.6	26.8	96.48	12	2
6	27	27	2	10	20	45	3
7	9	10	1.8	4.5	8.1	20	2
8	33	47					
9	22	35	1.5	0.3	0.45	1	1
10	30	33	1.2	0.3	0.36	11	1
11	11	11	0.6	0.0001	0.00006	0.001	0.0001
12	14	16	1.1	41	45	37	4
13	22	22	3	0.0001	0.0003	0.0001	0.0001
14	24	27	4.5	22	99	73	1
15	19	23	2.4	1.4	3	43	1
16	26	26					
17	12	17	2.1	0.0001	0.0001	0	0
18	11	18	1.5	4.2	6,3	15	8
19	10	11	3.5	0.01	0.035	0.0001	0.0001

*BTV* bi-testicular volume, *SA* semen analysis/seminal fluid examination at the end of the induction

We found no significant differences in BTV at 18 or 24 months of gonadotropin-mediated pubertal induction according to previous testosterone priming or pre-treatment with FSH (*p* = 0.2–0.38).

**Fig. 1:**
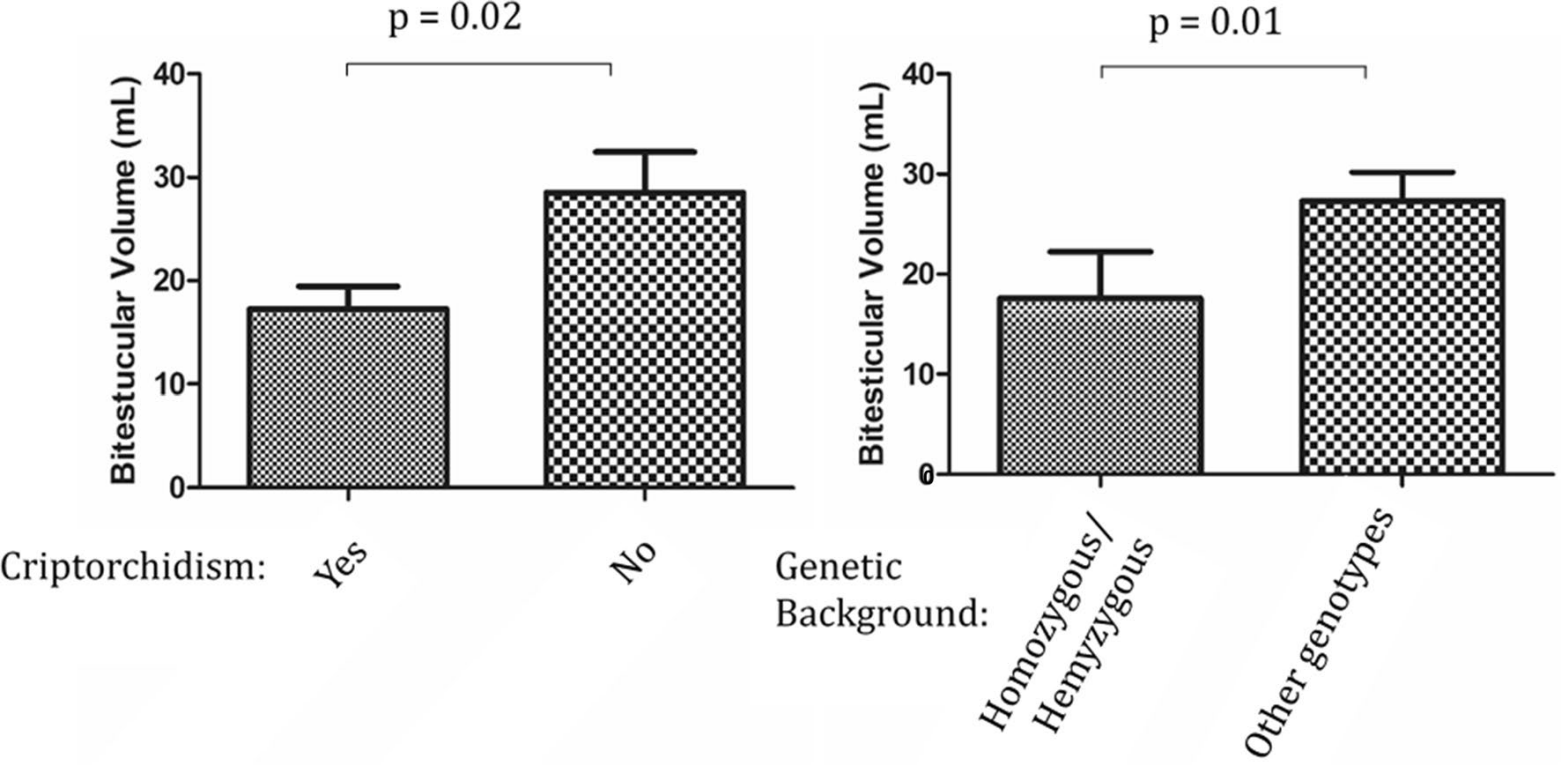
Comparison of bi-testicular volume after 24 months of treatment according to cryptorchidism and genotype. Other genotypes include both heterozygous and wild type patients

In contrast, BTV after 18 and 24 months of gonadotropin mediated pubertal induction resulted to be significantly (*p* = 0.04 and *p* = 0.05) associated with the presence of cryptorchidism at birth, as patients reporting a history of undescended testicle had smaller BTV (Fig. 1). No significance was achieved when only bilateral cryptorchidism was considered.

Mann Whitney analyses showed a highly significant correlation of gonadotropin-stimulated BTV with the presence of homozygous or hemizygous variants compared to heterozygous or WT patients (*p* = 0.007 and *p* = 0.01 at 18 or 24 months, respectively) (Table 1).

The response of spermatogenesis at the end of the induction was heterogeneous and did not show any significant correlation with the above-mentioned predictors.

Multiple linear regressions investigating together all the associated factors and the cumulative dose of hCG administered in 24 months, confirmed a significant correlation of smaller BTV after treatment with cryptorchidism (*p* = 0.02), complete forms of CHH (*p* = 0.03) and a tendency to associate with homozygous or hemizygous variants (*p* = 0.09) (Fig. 1). This tendency was again significant (*p* = 0.02), once considering as positive genetic background also variants found in oligogenicity and heterozygous mutations found in genes known to be autosomal dominant (AD), in addition to homozygous or hemizygous ones.

When evaluating the non-reproductive outcomes of gonadotropins-mediated induction, multiple regression analyses showed a significant association of eunuchoid habitus with both the age at the beginning of the induction (*p* = 0.04) and the duration of growth during induction (*p* = 0.029), but not with bone age before the induction, and cumulative hCG dose.

Performing the same multiple regression using the dependent variable the deltaSDSth, we found the same correlations with the age at the induction (*p* = 0.025), the duration of growth during therapy (*p* = 0.027), but also with the bone age at diagnosis (*p* = 0.031). Indeed, using simple regression, the ratio of arm span/height of the subject (a continuous variable related to eunuchoid habitus) was strongly correlated with deltaSDSth. No significant association was found with the cumulative dose of administered hCG in two years. We found no factor associated with greater penile length at 24th month of induction.

Studying the possible determinants of the duration of growth during the induction therapy, we found it to be associated with testosterone priming (*p* = 0.04) and to have a tendency to correlate with partial forms of CHH (defined as BTV at diagnosis > 8 mL; *p* = 0.053) with shorter growth span in patients with partial puberty at the diagnosis and in those having undergone testosterone therapy.

Kruskal–Wallis tests used to investigate if total testosterone values, PSA values, SDS height change from previous evaluation, growth rate, growth in BTV, increase in penile length, were associated with a specific weekly dosage of gonadotropins (and specifically to hCG) found that: (1) as expected T levels were strictly associated with the weekly dosage of hCG (*p* < 0.0001); (2) PSA also showed a trend to significance in the association with the weekly dosage of hCG (*p* = 0.06); (3) the administration of hCG, but not the dosage used, was found to be associated with a significant increment of height (*p* = 0.001); (4) the administration of hCG, but not the dosage used, was found to be associated to a significant increment of BTV (*p* = 0.002) and penile length (*p* = 0.007) after 6 months.

## Discussion

With the present study, we add a significant experience on the use of gonadotropins for pubertal induction. In particular, our cohort is composed only of male patients affected with isolated CHH, thus letting us evaluate (for the first time) the effect of the genetics underlying this rare condition, in the response to the treatment. We also studied all the clinical parameters that could predict a different response to gonadotropin treatment, both regarding reproductive and non-reproductive outcomes.

The determinants found associated with a smaller BTV at 24 months of gonadotropin stimulation were cryptorchidism, complete forms of CHH (BTV < 8 mL at diagnosis), and a positive genetic background, including homozygous, hemizygous, oligogenic and AD inherited conditions.

**Fig. 2:**
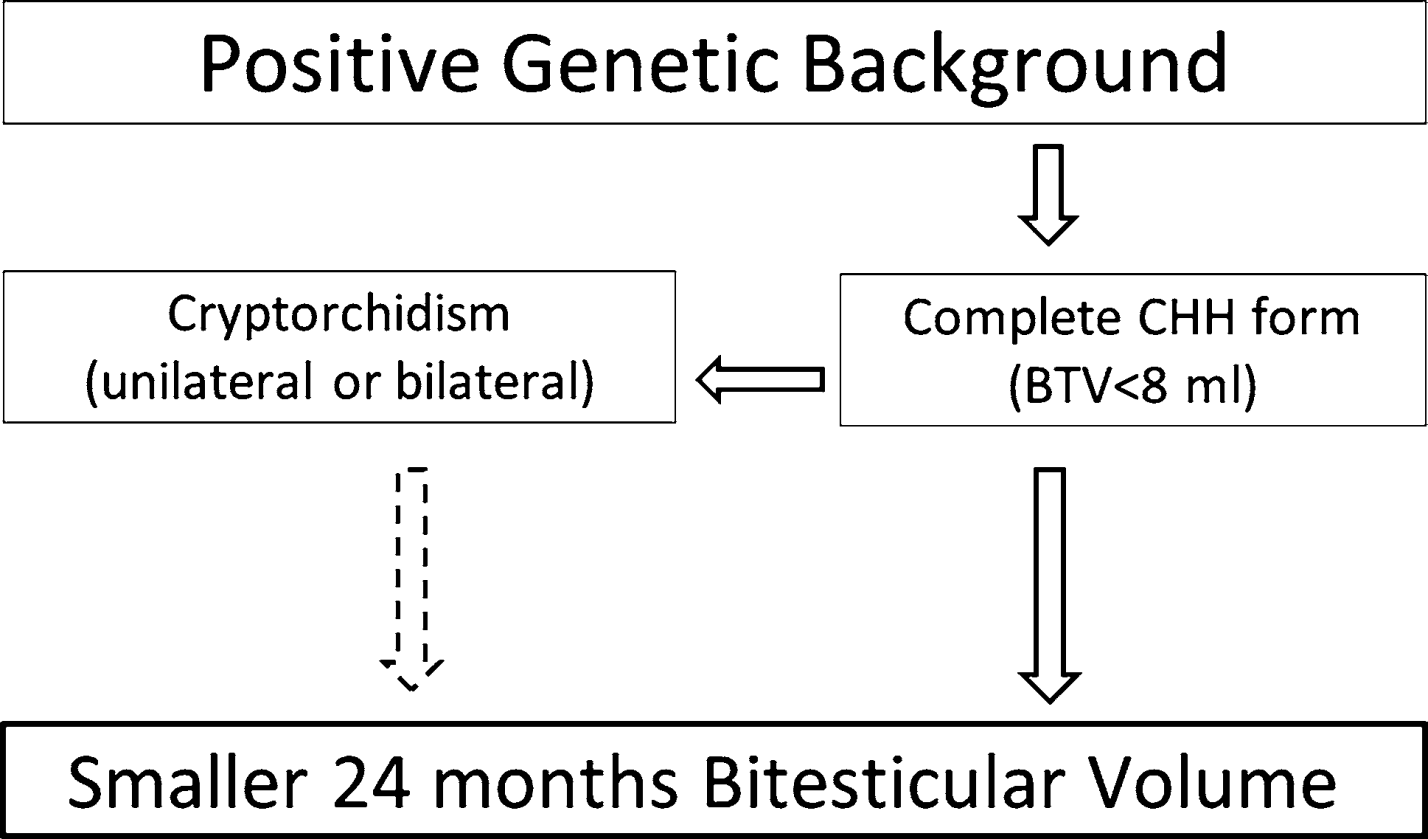
Hypothesis regarding the predictors of testicular response to gonadotropin-mediated induction

The relationship between cryptorchidism and smaller BTV [10] or poorer semen quality [16, 21] at the end of the induction was previously attributed to a protracted damage in the undescended testicles. However, it is known that cryptorchidism, especially bilateral forms and in association with micropenis and/or hypospadias, could rather be a sign of the complete lack of fetal/neonatal activation of HPG axis. Since in our cohort the relation between gonadal outcomes of the induction and cryptorchidism appears to persist even considering unilateral forms, we propose that the lower BTV at the end of the induction in cryptorchid patients is mainly related to the underlying cause, rather than due to a cause-effect relationship (Fig. 2). The lack of a gonadal stimulation by gonadotropins in early pre- or post-natal life can prevent testicle descent, and, probably, also an effective response to the exogenous gonadotropin stimulation later in life. Patients without cryptorchidism are more likely to have experienced at least a partial activation of the gonadal axis in early stages of life which could be necessary for the correct development of testes/Sertoli cells, to respond to gonadotropin-mediated pubertal induction.

Accordingly, homozygous and hemizygous variants in candidate CHH genes are significantly associated with smaller BTV at the end of the induction since are known to have high expressivity. Interestingly, the adjusted model using a multiple regression analysis showed a positive genetic background to be significantly associated with the testicular outcome of gonadotropin-mediated induction even when considering oligogenic forms of CHH and heterozygous variants in autosomal dominant genes. This last correlation can be explained by the usual association of homozygosis, hemizygosis or oligogenic transmission with a complete form of CHH, and the great disruptive potential of some AD genes leading to a defective minipuberty and fetal gonadotropin secretion. This further supports the importance of these early phases of gonadal axis development for the testicular maturation in later stages of life [3, 22–24] (Fig. 2). In contrast, the simple finding of a rare CHH variant was not associated with treatment outcomes. If many genes found harboring heterozygous allelic variants were once believed to have dominant transmission producing a full phenotype, nowadays we know that only the most disrupting ones have a true AD inheritance (i.e. FGFR1, CHD7), and the vast majority of pre(peri)-pubertal CHH is more likely to arise with hemizygosis (ANOS1), homozygosis, compound heterozygosity, or oligogenicity [1]. While mild/adult-onset forms of isolated HH (sometimes even in patients undergoing rehabilitation for obesity) have been associated to an enrichment in whatsoever CHH related rare allelic variant [18], pre-pubertal complete CHH forms, with higher expressivity and penetrance, can be explained only by severe, often combined, genetic defects.

The lack of significant associations of patients’ SA with all the above-mentioned factors, is probably due to the lower number of subjects undergoing the analysis, which also has high intra-individual variability.

As for the effects of previous treatments, we could not find any significant difference in the reproductive outcomes based on a pretreatment with FSH or with testosterone. In 2013, a study conducted on 13 HH patients induced with GnRH, which could be preceded or not by a FSH-only pretreatment [13], suggested that the FSH pre-treatment may be associated with better reproductive outcomes. Patients pretreated with FSH showed higher levels of inhibin B and greater proliferation and maturation of Sertoli cells as proved with a testicular biopsy and, even if no significant differences were recorded, tended to have greater testicular growth and better sperm production compared to the counterpart not undergoing FSH priming. These data suggested that FSH pre-treatment (mimicking the physiological FSH peak preceding the LH increase) could be relevant to prime Sertoli cells and maximize the following androgen-induced tubular maturation. The failure to find the same results in our retrospective analysis may be due to the lack of randomization and low numerosity; if controlled trials are surely needed, the present results might be precious in future metanalyses.

Evaluating previous testosterone therapies, we found no statistically significant difference in 18 and 24 months BTV between patients who were previously treated with testosterone and those who were not. Similar results were found in a prospective multicentric study comparing gonadotropin treatment in 27 prepubertal hypogonadotropic hypogonadal patients (naïve for every treatment) and in 23 already virilized patients who completed the induction with exogenous testosterone enanthate; however, even though there was no difference in final BTV, those authors found a (still underpowered) difference in semen analysis parameters with a higher prevalence of naïve subjects having a sperm count higher than 15 million/mL [16]. These data from the literature suggest a negative role of a previous pubertal induction therapy completed with exogenous testosterone, which we could not prove in our series. However, since all our patients who had a previous testosterone treatment did not complete the induction using testosterone (only used to exclude a constitutional delay of growth and puberty, CDGP), it is legitimate to suppose a minor effect.

Studying the non-reproductive outcomes of the therapy, it emerged that the strongest associations with a eunuchoid habitus are the chronological age at the beginning of treatment and the duration of growth during the gonadotropin induction, probably due to the classic delay in the diagnosis of CHH patients. The same associations, plus the one with bone age before the treatment, were found studying a measure of the effect of the disease and its treatment on the adult height of the subject (expressed as deltaSDSth). In fact, the eunuchoid habitus is strongly associated with deltaSDSth, with eunuchoid patients being higher than their target. This expected result confirms that their prolonged growth, due to a late growth plate fusion, produces both disproportioned longer limbs and (consequently) higher stature.

**Fig. 3:**
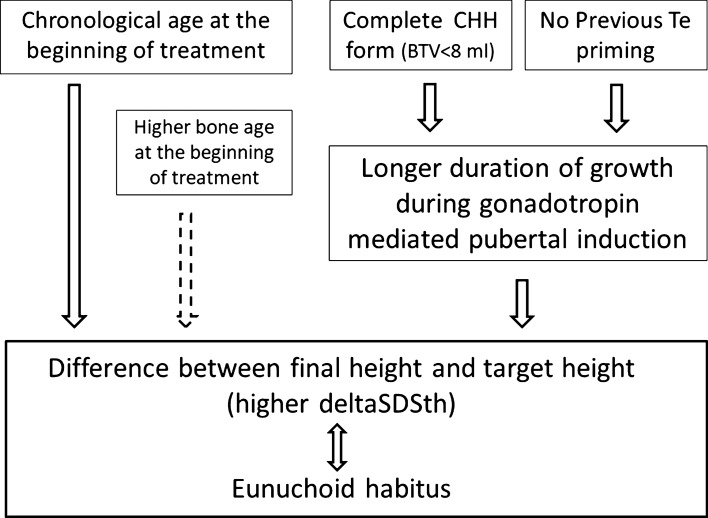
Hypothesis regarding the non-reproductive consequences of the induction with gonadotropins

It has been recently found that CHH patients are higher than general population and siblings [25], and our results confirm this first finding; however, we also found a shorter growth span (duration of growth during therapy) in patients with partial puberty at the diagnosis and in those having undergone testosterone priming, thus configuring the hypothesis illustrated in Fig. 3.

In fact, if the duration of growth during the induction therapy is usually longer than needed, especially in complete forms of CHH, previous testosterone priming, or partial activation of the axis, seem to mitigate this effect of adult height and disproportions.

This new notion of a lesser degree of disproportion in patients with previous testosterone priming or partial forms of CHH should be further investigated to understand if different treatment protocols are advisable according to age and these characteristics. Interestingly, opposite results on androgen priming were recently published by our group in CDGP [26]. These data indicate that an accurate and timely differential diagnosis between CDGP and CHH and treatment are crucial to gain height, and to avoid disproportionate features after puberty induction.

Kruskal–Wallis analysis, targeted to understand if specific dosages of gonadotropins are related to major changes, did not give any particular result. This is probably due to the retrospective design of the study, and that usually higher dosages were given later during the induction, thus preventing us from separating the effect of the dosage from the effect of a prolonged treatment.

As expected, T levels highly correlate with the weekly dosage of hCG, whereas we found no significant difference in hematocrit values, highlighting the safety of this approach. PSA (androgenization index), on the contrary, showed a tendency to correlate with hCG weekly dosage. The only significant change of growth in height, testicular volume and penile length was found when hCG was added, thus confirming that they are secondary to testosterone-induced androgenization.

Concluding, our retrospective analysis shows that positive genetic background, accounting for complete CHH forms, may be a relevant factor preventing an optimal testicular response to gonadotropin stimulation in young CHH patients. Prolonged gonadotropin stimulation or a post-natal priming of the gonadal axis may be needed to obtain satisfactory results on testicular maturation at peri-pubertal or adult ages in complete CHH forms. Moreover, our data suggest the effect of a timely androgenization (also with short-term testosterone priming for initial management and differential diagnosis of CHH) on body proportions. Large multicentric, RCT are needed to confirm these evidences.

## Supplementary Information

Below is the link to the electronic supplementary material.

Supplementary file1 (DOCX 16 KB)

## Data Availability

The data will be uploaded and available upon reasonable request.
