# Supplementary material for: Predictors of reproductive and non-reproductive outcomes of gonadotropin mediated pubertal induction in male patients with congenital hypogonadotropic hypogonadism (CHH)
Source: J Endocrinol Invest. 2021 Mar 18;44(11):2445–54. doi: 10.1007/s40618-021-01556-x (PMC8502167; doi:10.1007/s40618-021-01556-x)
Supplement: Supplementary file 1 — Supplementary file1 (DOCX 16 KB) [file 40618_2021_1556_MOESM1_ESM.docx]

Supplementary Table 1. Indicative scheme of pubertal induction used. Dosages were tailored for each patient according to chronological and bone age at diagnosis.

| **Time** | **Phase of induction** | **FSH**  **IU** | **hCG**  **IU** | **Monitoring** |
| --- | --- | --- | --- | --- |
| 0 | FSH pre-treatment | 75x3 | - | TANNER, BTV, Hormonal evaluation, HCT, PSA |
| 4 months | Starting hCG | 75x3 | 250x3 | TANNER, BTV, Hormonal evaluation, HCT, PSA |
| 10 months | 6 months hCG | 75x3 | 500x2 | TANNER, BTV, Hormonal evaluation, HCT, PSA |
| 16 months | 12 months hCG | 75x3 | 500x3 | TANNER, BTV, Hormonal evaluation, HCT, PSA |
| 22 months | 18 months hCG | 75x3 | 1000x2 | TANNER, BTV, Hormonal evaluation, HCT, PSA |
| 28 months | 24 months hCG, end of induction | Switch to testosterone | | TANNER, Hormonal evaluation, HCT, PSA Semen Analysis, BTV, testicular US |
